# Emergency Department Treatment Provides Immediate and Durable Relief Following Vaccine Injury: A Case Report

**DOI:** 10.5811/cpcem.2022.11.57642

**Published:** 2023-01-24

**Authors:** Adam Rowh, Marta Rowh, Mark Goodman

**Affiliations:** * University of Colorado Health System, Department of Emergency Medicine, Loveland, Colorado; † St. Charles Health System, Department of Emergency Medicine, Bend, Oregon; ‡ University of Colorado School of Medicine, Department of Emergency Medicine, Aurora, Colorado

**Keywords:** vaccine, shoulder injury related to vaccine administration (SIRVA), mass vaccination, ultrasound, case report

## Abstract

**Introduction:**

Intramuscular administration of vaccines into the deltoid muscle is the recommended route for most vaccines in adults. Ectopic injection into the subdeltoid/subacromial bursa can produce an inflammatory bursitis that is associated with significant long-term morbidity.

**Case Report:**

We describe a novel approach to treatment of this condition: ultrasound-guided administration of dexamethasone by the emergency physician within six hours of vaccine administration. This approach resulted in complete and durable long-term resolution of symptoms with no functional impairment.

**Conclusion:**

This outcome is superior to that described for usual care, and the approach is well-suited to emergency physicians.

## INTRODUCTION

Routine immunization, when performed correctly, is extremely safe and effective at preventing numerous diseases. Intramuscular injection into the deltoid is the recommended route of administration for most vaccines in adults.^1^ Inadvertent administration of a vaccine dose into the subacromial/subdeltoid bursa can produce a syndrome of severe inflammatory bursitis that can have significant long-term consequences.^2^–^5^ Identification of this condition is typically delayed, and treatment, administered weeks to months after the vaccination, is not reliably curative.^3^

In this case report, we describe a novel approach to this condition. An 83-year-old female presented to the emergency department (ED) with suspected shoulder injury related to vaccine administration (SIRVA) following administration of a seasonal influenza vaccine. She was treated by the emergency physician with ultrasound-guided corticosteroid injection within six hours of the vaccination. In contrast with the prolonged and incomplete recovery typically described, this patient experienced complete resolution of pain and restored range of motion within 24 hours. The patient remained asymptomatic at three-month follow-up.

## CASE REPORT

An 83-year-old woman was administered an unspecified influenza vaccine into her right upper arm during a routine visit to her primary care office. She presented to the emergency department (ED) approximately four hours thereafter out of concern for severe and progressive pain in the shoulder. Her pain was located in the shoulder joint globally and was exacerbated by movement. She reported that the pain began within two hours after the immunization was administered, and she stated that her symptoms were atypical compared to her lifelong history of other routine immunizations. Specifically, she described an immediate pain in the soft tissues that was familiar and unconcerning, reminiscent of prior intramuscular injections, and which resolved within minutes. Subsequently, a deeper and more poorly localized pain began gradually after she had left the doctor’s office. This increased until she presented to the ED. She had no significant past medical history, including no prior history of musculoskeletal or rheumatologic disorders.

She had normal vital signs with no fever. Examination of her shoulder demonstrated no visible external abnormality except for the punctate wound of the vaccine needle, which was located approximately one centimeter inferior to the acromion process. There was no warmth or surrounding erythema, and there was only minimal soft tissue tenderness at the vaccine site. Active and passive range of motion were equally limited and painful in all planes of motion at the glenohumeral joint. Sensory and motor function were intact throughout the extremity in the axillary, radial, median, and ulnar nerve distributions. Perfusion was normal. She had no abnormalities of the contralateral shoulder or the remainder of her physical examination.

Bedside ultrasound was performed, which demonstrated enlargement of the subacromial/subdeltoid bursa and no effusion of the glenohumeral joint. We consulted orthopedics and discussed the case. After review of the findings, the emergency medicine and orthopedic surgery attending physicians agreed that offering corticosteroid injection of the affected bursa was appropriate. After a shared decision-making conversation with the patient, involving discussion of the reasonable therapeutic alternatives, she expressed her consent and desire to proceed. Using real-time ultrasound guidance, 6 milligrams (mg) of dexamethasone was injected into the abnormally fluid-filled bursa along with 10 mg of bupivacaine. She experienced significant, but incomplete, relief after an additional 60 minutes of observation in the ED. Upon follow-up 24 hours after discharge, she reported that her symptoms had completely resolved. She had no further pain, and her range of motion had returned to normal. Follow-up at three months confirmed that she had no return of her initial symptoms, and the function and range of motion of her shoulder were normal.

## DISCUSSION

In typical anatomy, the subacromial and subdeltoid bursae are a single communicating structure that extends lateral to the acromion by 3–6 centimeters (cm) and lies 0.8–1.6 cm beneath the skin surface.^3^,^6^ As a result of this anatomic location, the bursa is susceptible to inadvertent injury during routine immunization when technique is incorrect. Bodor and Montalvo^7^ described a specific syndrome of prolonged shoulder pain and impaired function observed after routine immunization, distinct from the typical short-lived discomfort commonly experienced. Atanasoff et al named the syndrome shoulder injury related to vaccine administration (SIRVA) following identification of cases in the Vaccine Injury Compensation Program database.^3^ Further research has suggested that antigen deposition in the shoulder bursa is the likely mechanism for the exaggerated and prolonged bursitis that results.^8^ This syndrome has been observed following multiple types of vaccine.^2^,^9^–^11^ It is preventable by employing correct injection technique.^1^,^12^

*CPC-EM Capsule*

What do we already know about this clinical entity?

*Injection of vaccine into the shoulder bursa can cause long-lasting pain and disability. Recognition is usually delayed, and current therapies provide only partial relief.*

What makes this presentation of disease reportable?

*This is the first report of this condition being recognized and treated by emergency physicians. The treatment described here provided relief superior to what is described elsewhere.*

What is the major learning point?

*Early recognition and intervention on this condition may prevent long-term symptoms. The treatment described here is simple and should be understood by emergency physicians.*

How might this improve emergency medicine practice?

*Emergency physicians may be ideally suited to identifying and treating this condition. Early intervention appears to maximize recovery from this uncommon but debilitating injury.*

Clinical features of SIRVA include unusually severe pain in the shoulder within several hours of vaccine administration, followed by restricted range of motion.^13^ The natural history involves prolonged pain and dysfunction, typically lasting weeks to months and occasionally without complete resolution.^9^,^10^ Treatment for this entity specifically is not well described but generally includes physical therapy, steroid injections, and surgical procedures.^14^–^16^ One case series was published describing the early (ie, within five days of vaccination) use of steroid injections.^16^ These authors observed resolution of symptoms more quickly than anticipated compared to other published reports, and proposed corticosteroid injection as a plausible first-line intervention when this diagnosis is suspected.

## CONCLUSION

This is the first published case of corticosteroid injection performed by an emergency physician for shoulder injury related to vaccine administration. The literature supports the proposed mechanism of SIRVA: ectopic vaccine administration causing acute, immune-mediated inflammatory bursitis. The immunologic response propagates after exposure to antigen, suggesting that delay in treatment is likely to yield decreased effectiveness. Early (within five days) treatment has been observed to be effective, but no randomized controlled trials exist given the rarity of the event. Minimizing the delay before immunomodulatory intervention would be expected to provide the most effective prophylaxis following identification of possible SIRVA. This can be achieved by administering a steroid dose as early as possible, an approach that is amenable to being performed in an ED.

**Image 1:**
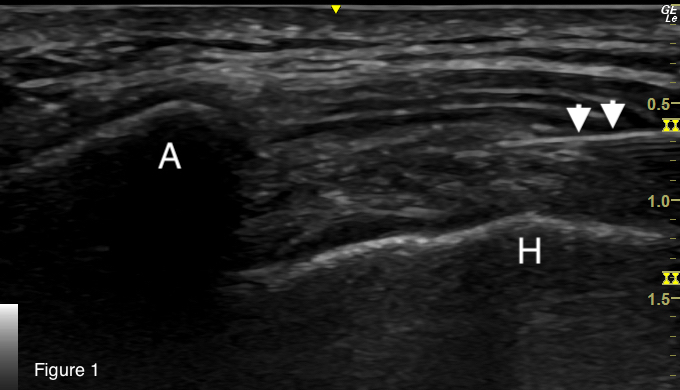
Ultrasound still image demonstrating ultrasound-guided approach to injection of the subacromial/subdeltoid bursa. (A: acromion; H: humeral head; arrowheads: needle.)

**Image 2:**
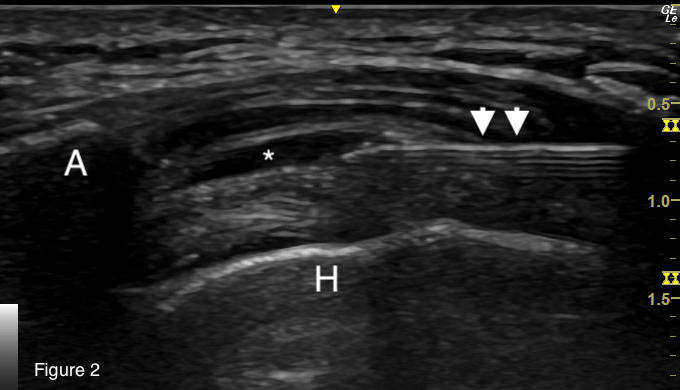
Ultrasound still image demonstrating injection into the subacromial/subdeltoid bursa. (A: acromion; H: humeral head; arrowheads: needle; asterisk: subacromial/subdeltoid bursa distended with injectate.)

Although orthopedic injections are an infrequent component of the routine practice of emergency medicine, they are entirely within the scope of an emergency physician’s training and expertise. Ultrasound guidance is preferred for greater procedural accuracy (see Image 1 and Image 2),^17^ but landmark approaches to corticosteroid injections of this bursa are also described.^18^ Offering this intervention in the ED to patients with suspected SIRVA provides access to a potentially disease-modifying treatment that would otherwise be delayed until outpatient specialist consultation can be arranged, a delay that is plausibly associated with a decreased efficacy of the intervention.

Vaccination will remain a cornerstone of public health disease-mitigation strategy, and with the advent of mass-vaccination initiatives during the COVID-19 pandemic, vaccines are likely to be administered by increasing numbers of healthcare personnel with more variation in training and experience. In this context, SIRVA may become more common. Early recognition and prompt intervention have the potential to significantly reduce the long-term morbidity associated with this condition. Further research is indicated to validate and standardize the approach presented here for widespread implementation.
